# Dentists of Maryland and Washington Meet

**Published:** 1894-05

**Authors:** 


### Editorial, Etc.

DENTISTS OF MARYLAND AND WASHINGTON MEET.—The Maryland State Dental Association and the Washington City Dental Society held a joint meeting in Washington April 24 and 25, 1894, after an interval of eleven years. The committee of arrangements for the meeting was H. M. Schooley of Washington, chairman; Cyrus W. Gingrich of Baltimore, secretary; William Donnally, J. Roland Walton, F. F. Drew and C. C. Harris. The sessions were held at the law school of the National University.

The programme was as follows: Morning session, Tuesday at 10 A. M.—Opening address by chairman of joint committee, Dr. H. M. Schooley; address of welcome by president of Washington Dental Society, Dr. W. E. Dieffenderfer; response by president of the Maryland State Dental Association, Dr. B. Holly Smith of Baltimore; report of committee on publication, voluntary essays and dental legislation, Dr. M. F. Finley of Washington.

At the afternoon session, report of committee on dental education, literature and dental nomenclature, Edward Nelson, of Frederick, chairman. Clinics, under direction of William Donnally, D. D. S., Washington.

Evening session, 8 p. m., report of committee on mechanical dentistry and dental chemistry, Dr. J. B. Hokgkin of Washington. Essay, "Uric acid and the Dental Diseases of the Gouty Diathesis," Dr. L. Ashley Faught of Philadelphia, Pa. Report of committee on pathology and therapeutics, Dr. J. Wilson Davis of Washington.

Wednesday morning session, report of committee on operative dentistry and surgery, Dr. Thomas H. Davy, of Baltimore, chairman. Essay, "Dental Legislation," Dr. A. W. Sweeney, of Washington; address, Dr. W. G. A. Bonwill of Philadelphia.

Afternoon session, 2 p. m., report of committee on anatomy, physiology and histology, Dr. R. B. Winder of Baltimore. Clinics, 3 p. m., under the direction of Dr. William Donnally of Washington.

Evening session—Report of committee on orthodontia and dental appliances, Dr. William Donnally of Washington. Banquet at 8 p. m., Dr. M. W. Foster, of Baltimore, presiding.

Clinics—"Bleaching Teeth by Use of Pyrozone," Dr. Charles A Meeker, Newark, N. J.; "Correction of Irregularities" (models and appliances exhibited), Dr. V. H. Jackson, New York; "Methods of Taking Impressions" (with demonstrations, etc.), Dr. S. G. Watkins, Montclair, N. J.; "Combination Gold Fillings" (cohesive and non-conesive foil), Dr. A. Lee Penuel, Leesburg, Va.; "Gold Filling (Steurer's gold in connection with cohesive gold), Dr. Harvey Iredell, New Brunswick, N. J.; "Non-cohesive Gold Filling" Dr. S. J. Cockerille, Washington; "A Method of Securing Perfect Surface Fac similes of the Speyer Cohesive Forms as Applicable to Vulcanic Plates," Dr. F. C. Barlow, D. D. S., Jersey City; "Combination of Gold and Tin Fillings," Dr. S. G. Watkins, Montclair, N. J.; "Irregularities; Practical Cases Showing Changes in Facial Contour," Dr. C. S. Case, Chicago.

Later the Maryland State Dental Association has elected the following officers: Dr. C. C. Harris, president; Dr. F. F. Drew, first vice-president; Dr. M. G. Sykes, second vice-president; Dr. W. W. Dunbracco, recording secretary; Dr. A. Price, corresponding secretary, and Dr. J. G. Heiusler, treasurer. Executive committee: Drs. Cyrus M. Gingrich, W. S. Twilley and W. B. Finney.

